# Substance Use From Social Distancing and Isolation by US Nativity During the Time of COVID-19: Cross-sectional Study

**DOI:** 10.2196/38163

**Published:** 2023-02-17

**Authors:** Francisco Alejandro Montiel Ishino, Kevin Villalobos, Faustine Williams

**Affiliations:** 1 Division of Intramural Research National Institute of Environmental Health Sciences National Institutes of Health Durham, NC United States; 2 Division of Intramural Research National Institute on Minority Health and Health Disparities National Institutes of Health Bethesda, MD United States

**Keywords:** substance use, COVID-19, US nativity

## Abstract

**Background:**

The COVID-19 pandemic had many unprecedented secondary outcomes resulting in various mental health issues leading to substance use as a coping behavior. The extent of changes in substance use in a US sample by nativity has not been previously described.

**Objective:**

This study aimed to design a web-based survey to assess the social distancing and isolation issues exacerbated by the COVID-19 pandemic to describe substance use as a coping behavior by comparing substance use changes before and during the pandemic.

**Methods:**

A comprehensive 116-item survey was designed to understand the impact of COVID-19 and social distancing on physical and psychosocial mental health and chronic diseases. Approximately 10,000 web-based surveys were distributed by Qualtrics LLC between May 13, 2021, and January 09, 2022, across the United States (ie, continental United States, Hawaii, Alaska, and territories) to adults aged ≥18 years. We oversampled low-income and rural adults among non-Hispanic White, non-Hispanic Black, Hispanic or Latino, and foreign-born participants. Of the 5938 surveys returned, 5413 (91.16%) surveys were used after proprietary expert review fraud detection (Qualtrics) and detailed assessments of the completion rate and the timing to complete the survey. Participant demographics, substance use coping behaviors, and substance use before and during the pandemic are described by the overall US resident sample, followed by US-born and foreign-born self-reports. Substance use included the use of tobacco, e-cigarettes or nicotine vapes, alcohol, marijuana, and other illicit substances. Marginal homogeneity based on the Stuart-Maxwell test was used to assess changes in self-reported substance use before and during the pandemic.

**Results:**

The sample mostly included White (2182/5413, 40.31%) and women participants (3369/5406, 62.32%) who identified as straight or heterosexual (4805/5406, 88.88%), reported making ≥US $75,000 (1405/5355, 26.23%), and had vocational or technical training (1746/5404, 32.31%). Similarities were observed between the US-born and the foreign-born participants on increased alcohol consumption: from no alcohol consumption before the pandemic to consuming alcohol once to several times a month and from once to several times per week to every day to several times per day. Although significant changes were observed from no prior alcohol use to some level of increased use, the opposite was also observed and was more pronounced among foreign-born participants. That is, there was a 5.1% overall change in some level of alcohol use before the pandemic to no alcohol use during the pandemic among foreign-born individuals, compared with a 4.3% change among US-born individuals.

**Conclusions:**

To better prepare for the inadvertent effects of public health policies meant to protect individuals, we must understand the mental health burdens that can precipitate into substance use coping mechanisms that not only have a deleterious effect on physical and mental health but also exacerbate morbidity and mortality in a disease like COVID-19.

## Introduction

### Background

The COVID-19 pandemic, as of March 2022, has >456 million recorded cases and 6 million reported deaths worldwide [1]. Although COVID-19 is caused by SARS-CoV-2, increased morbidity and mortality are associated with multiple direct and indirect social, physiological, and environmental factors [2,3]. There were, however, indirect health effects of the pandemic that affected mental health [4] and exacerbated maladaptive coping mechanisms such as substance use [5,6].

Coping strategies provide the ability to manage external and internal demands, given an individual’s resources [7]. When demands are exceeded owing to high levels of physiological and psychological stress, as observed during the COVID-19 pandemic, it can lead to the adoption of maladaptive coping behaviors [8,9]. As such, mental health issues were exacerbated by multiple social and environmental stressors, social distancing, and isolation during the COVID-19 pandemic that in turn affected coping mechanisms leading to changes in substance use patterns [10,11].

Statistics for June 2020, when compared with 2019, as reported by the Centers for Disease Control and Prevention [5], show that 13% of US adults aged ≥18 years started or increased substance use to cope with stress from the COVID-19 pandemic. Reports of increased use were found for both licit and illicit substances [12,13] such as alcohol [14] and opioids [15], respectively. There were also mixed findings regarding the use of substances such as tobacco [16-18] and marijuana [19] as well as their respective methods of use through vaping [13,20,21]. Moreover, using licit and illicit substances can cause increased morbidity and mortality, especially when considering the physiological effects of COVID-19, in addition to worsening mental health [5].

The effects of social distancing and isolation early in the pandemic were compounded by multiple issues associated with access to health, mental health, and related telehealth services [22]. Multiple deficits and barriers to mental health services, especially among underserved and underrepresented communities, precipitated health care access and treatment disparities [6,10,23]. These deficits and barriers were then exacerbated by the COVID-19 pandemic and may have led to substance use as a prevalent coping mechanism during a period when substance treatment options were already limited [10,22,23]. Nonetheless, studies on substance use as a coping strategy during the COVID-19 pandemic are limited.

### Objectives

Further research is needed to understand the multiple and varying sociodemographic and socioeconomic factors to fill this gap. In addition, there is a critical need to include both US-born and foreign-born individuals in substance use research during the COVID-19 pandemic as these studies are still limited. A limited number of studies have reported mixed findings, that is, increased and decreased substance use during the pandemic [24,25]. Nevertheless, the psychological effects of substance use may be detrimental and synergize disparities among racial and ethnic foreign-born minorities [24,26,27]. As such, our purpose was to describe substance use as a coping mechanism to COVID-19–induced social distancing and isolation by comparing substance use before and during the pandemic by US-born and foreign-born individuals.

## Methods

### Overview

Our study *Understanding the Impact of the Novel Coronavirus (COVID-19) and Social Distancing on Physical and Psychosocial (Mental) Health and Chronic Diseases* created a comprehensive 116-item web-based survey that was nationally distributed in the United States. The survey modules included (1) general health status; (2) COVID-19 symptoms, testing, and prevention; (3) chronic illness management; (4) social distancing; (5) mental health; (6) pandemic economic impact; (7) discrimination; and (8) sociodemographics.

The target population comprised adults aged ≥18 years residing in the United States. The US resident sample included both US-born and foreign-born participants. Qualtrics LLC was contracted to facilitate the recruitment and distribution of the web-based survey to both US-born and foreign-born racial and ethnic groups. The US-born racial and ethnic groups included Hispanic or Latino, White, Black, Asian, American Indian and Alaskan Native, and Native Hawaiian and Pacific Islander participants. Foreign-born racial and ethnic groups included African, Middle Eastern, Hispanic or Latino, and Asian participants. Qualtrics then used proprietary consumer panels to randomly sample White participants that matched demographic characteristics with other racial and ethnic groups. We oversampled adults with low income (<US $25,000 annual household income) who resided in rural areas (self-reported and cross-referenced with zip codes already collected by Qualtrics) among non-Hispanic White, non-Hispanic Black, Hispanic, and foreign-born participants. The survey was available only in English.

A total of 10,000 surveys were distributed between May 13, 2021, and January 09, 2022. The initial surveys received by Qualtrics were assessed via expert review fraud detection to prevent multiple submissions and detect “bots” to protect the integrity of the data. After the assessment, 5938 surveys were received by the research team from Qualtrics. Information Management Services, Inc, a research support firm that provides analytic services, was given the task to clean and manage the deidentified survey data.

To improve study integrity, initial data cleaning by the Information Management Services included flagging surveys based on the completion rate and the timing to complete the survey. Participants were flagged and removed from the analysis if they completed <80% of the survey based on 102 questions after accounting for skip pattern items or took <5 minutes to complete the survey. In total, 125 surveys were removed at this stage, giving us 5813. Our study ended with a total of 5413 surveys based on the completed responses in the social distancing module of the *Understanding the Impact of the Novel Coronavirus (COVID-19) and Social Distancing on Physical and Psychosocial (Mental) Health and Chronic Diseases* survey. The survey can be requested from the principal investigator, Faustine Williams, PhD, MPH, MS, from the National Institute on Minority Health and Health Disparities of the National Institutes of Health (NIH) in Bethesda, Maryland.

### Ethical Considerations

Qualtrics recruited study participants, and web-based informed consent was provided before the survey. Participants were asked to participate in a voluntary research study titled *Understanding the Impact of the Novel Coronavirus (COVID-19) and Social Distancing on Physical and Psychosocial (Mental) Health and Chronic Diseases* conducted by Faustine Williams. If they chose to participate in the study, they were assured that their responses would be kept confidential. Participants were reminded that the study was voluntary; as such, they could change their minds at any time after starting the survey and opt out with no fear of repercussions. Participants could skip any questions that they did not want to answer. All answers were kept confidential, and Qualtrics assured the participants that no personal identifiers would be shared with the National Institute on Minority Health and Health Disparities research team. Participants were to receive an incentive of a US $10 gift card after the completion of their survey, which would take approximately 30 minutes of their time. At the end of the survey, participants were also asked if they would be interested in participating in another follow-up survey. Then, Qualtrics would follow-up with those who responded that they were interested. Participants were provided the contact information of the principal investigator (Faustine Williams) as well as the phone number of the NIH Institutional Review Board.

The research protocol for this study was reviewed by the NIH, Intramural Research Program Institutional Review Board, Human Research Protection Program, and Office of Human Subjects Research Protections and received an exemption on December 23, 2020 (IRB#000308). The NIH, Intramural Research Program Institutional Review Board, Human Research Protection Program, and Office of Human Subjects Research Protections determined that our protocol did not involve human participants and was excluded from the institutional review board review.

### Descriptors

#### Sociodemographic Variables

All sociodemographic items were self-reported and allowed for either the selection of multiple categories or provided a free response if they selected a blank or other category. Nativity was categorized by country of birth as either US born or foreign born. US-born nativity was based on respondents’ self-reported births in the 48 contiguous states, Washington, the District of Columbia, Alaska, Hawaii, and other US territories such as Puerto Rico. Foreign-born nativity was based on respondents’ self-reported births occurring in another country outside the United States based on the US birth classification. Racial and ethnic categories included selecting ≥1 of the following options: White, Black or African American, Asian, American Indian or Alaskan Native, Hawaiian or Pacific Islander, African, Middle Eastern, and multiracial or multiethnic. If respondents selected ≥2 racial or ethnic groups, they were classified as multiracial and multiethnic. Gender categories included men, women, nonbinary, transgender people, and others. Sexual orientation included straight or heterosexual, lesbian, gay, bisexual, and other. The lesbian and gay categories were combined. Age was self-reported starting from 18 to ≥85 years. Age categories were then constructed as follows: 18 to 35 years, 36 to 55 years, and 56 to ≥85 years. Annual household income was reported as <US $25,000, US $25,000 to US $34,999, US $35,000 to US $49,999, US $50,000 to US $74,999, and US ≥$75,000. Educational attainment was categorized by self-reported highest schooling that included (1) less than high school or General Language Development (ie, did not attend school; elementary education, 6 years or less; more than elementary to junior high school; or some high school), (2) high school diploma or General Language Development, (3) some college or vocational or technical schooling, (4) bachelor’s degree, and (5) master’s degree or above (ie, master’s degree or doctoral, professional, or postgraduate degree). Employment status was assessed using multiple survey items. Current employment (ie, employed, self-employed, unpaid or voluntary work, apprenticeship or vocational training, disabled, caretaker or looking after family or home, in school, retired, or unemployed) and if considered an essential worker (ie, no or yes). Unemployed or nontraditional work was categorized as being disabled, a caretaker or looking after family or home, in school, retired, or unemployed. Nonessential workers were categorized as not considered an essential worker and employed, self-employed, unpaid or voluntary work, or apprenticeship or vocational training. Essential workers were categorized as being considered an essential worker and employed, self-employed, unpaid or voluntary work, or apprenticeship or vocational training.

#### Substance Use

Coping behaviors for social distancing and isolation during the COVID-19 pandemic were assessed by asking questions regarding substance use in the social distancing module of the survey. Questions specific to exclusively using the following substances to cope were used: cigarettes or vaping, increased alcohol use, marijuana use, and illicit substance use. To assess cases of substance use during the pandemic, we asked “During the past month, how often did you” (1) smoke cigarettes or other tobacco products for tobacco use, (2) smoke e-cigarettes or other nicotine vaping products, (3) have a drink containing alcohol for alcohol use, (4) use marijuana, and (5) use illicit drugs. Illicit drugs were defined as other substances that were not previously listed (ie, tobacco, nicotine, alcohol, or marijuana) and could include but not be limited to opiates, hallucinogens, cocaine, or amphetamines. A follow-up control question was used for each aforementioned question category that asked, “Compared to before the pandemic, this is or was... .” Responses to each question had the following levels: not at all, once during the month, several times a month, once a week, several times a week, almost every day or every day, and several times a day. Responses were collapsed to (1) not at all, (2) once to several times per month, (3) once to several times per week, and (4) every day to several times per day.

### Analytic Procedure

Descriptive statistics of survey sample sociodemographics and substance use behaviors during the COVID-19 pandemic were assessed by the nativity of respondents, that is, US born and foreign born. Descriptives for the overall survey sample before and during the pandemic were assessed, followed by a more detailed assessment by nativity. A Stuart-Maxwell test was used to examine whether substance use before the COVID-19 pandemic was equal to substance use during the pandemic among survey respondents. If significant differences were found in substance use, we tested for differences based on participant self-reported nativity. The Stuart-Maxwell test is an ideal nonparametric test to examine asymptotic symmetry and marginal homogeneity on matched-pair controls (ie, before the COVID-19 pandemic) and cases (ie, during the COVID-19 pandemic) with various discrete levels of substance use (ie, not at all, once several times per month, once to several times per week, and every day to several times per day). All analytical procedures were conducted using Stata/MP (version 16.1; StataCorp LLC). All analytical files are available upon reasonable request.

## Results

### Overview

The overall sample was primarily racially and ethnically White, Black or African American, and Hispanic or Latin. Most of the participants self-reported as women (3369/5406, 62.32%); were straight or heterosexual (4805/5406, 88.88%); had a household annual income of US ≥$75,000 (1405/5355, 26.23%); had some college, vocational, or technical training (1746/5404, 32.31%); and were unemployed or participated in nontraditional work (2401/5405, 44.42%). The age of the sample was between 18 and 35 years (1839/5119, 35.92%) and 36 and 55 years (1971/5119, 38.5%). The sample self-reported primarily using the following substances in the following order based on proportion: marijuana use (109/5404, 2.02%), increased alcohol use (87/5404, 1.61%), e-cigarette or nicotine vape (83/5404, 1.54%), and illicit substance use (24/5404, 0.44%). A more detailed breakdown of the sociodemographic profiles and substance use by nativity is shown in Table 1.

Table 2 describes substance use before and during the COVID-19 pandemic for the overall sample. Most of the participants who reported substance use before the COVID-19 pandemic used tobacco every day to several times per day (940/5130, 18.32%), e-cigarettes or nicotine vapes once to several times per month (366/5130, 7.13%), alcohol once to several times per month (1563/5130, 30.47%), marijuana every day to several times per day (471/5130, 9.18%), and other illicit substances once to several times per month (225/5130, 4.38%). During the COVID-19 pandemic, the same participants used tobacco every day to several times per day (664/3802, 17.46%), e-cigarettes or nicotine vapes once to several times per week (269/3802, 7.07%), alcohol once to several times per month (1051/3802, 27.64%), marijuana every day to several times per day (336/3802, 8.84%), and other illicit substances once to several times per week (148/3802, 3.89%).

In Table 3, we describe substance use before and during the COVID-19 pandemic by US-born and foreign-born individuals. If reporting substance use before the COVID-19 pandemic, the US-born sample primarily reported use of tobacco every day to several times per day (846/3903, 21.68%), e-cigarettes or nicotine vapes once to several times per week (298/3889, 7.66%), alcohol once to several times per month (1207/3903, 30.92%), marijuana every day to several times per day (420/3873, 10.84%), and other illicit substances once to several times per week (196/3887, 5.04%). The same US-born sample during the COVID-19 pandemic, if reporting substance use, used tobacco every day to several times per day (600/2912, 20.60%), e-cigarettes or nicotine vapes once to several times per week (231/2899, 7.97%), alcohol once to several times per month (812/2921, 27.80%), marijuana every day to several times per day (300/2901, 10.34%), and other illicit substances once to several times per week (131/2896, 4.52%).

The foreign-born sample’s substance use before the COVID-19 pandemic indicated tobacco use every day to several times per day (94/1179, 7.97%), e-cigarettes or nicotine vapes once to several times per month (75/1176, 6.38%), alcohol once to several times per month (356/1187, 29.99%), marijuana once to several times per month (62/1170, 5.3%), and other illicit substances once to several times per month (44/1180, 3.73%). The same foreign-born sample during the COVID-19 pandemic, if reporting substance use, used tobacco every day to several times per day (67/860, 7.79%), e-cigarettes or nicotine vapes once to several times per week (39/857, 4.55%), alcohol once to several times per month (242/860, 28.14%), marijuana once to several times per month (41/855, 4.79%), and other illicit substances once to several times per month (24/852, 2.82%).

**Table 1:** Sample descriptives.

Participant characteristics		US born (n=4166), n (%)	Foreign born (n=1247), n (%)	Total (n=5413), n (%)
**Race and ethnicity (n=5413)**				
	White	2053 (49.28)	129 (10.34)	2182 (40.31)
	Black or African American	1024 (24.58)	181 (14.51)	1205 (22.26)
	Hispanic or Latino	551 (13.23)	435 (34.88)	986 (18.22)
	Asian	217 (5.21)	338 (27.11)	555 (10.25)
	American Indian or Alaska Native	137 (3.29)	8 (0.64)	145 (2.68)
	Hawaiian or Pacific Islander	47 (1.13)	14 (1.12)	61 (1.13)
	African	28 (0.67)	26 (2.09)	54 (1)
	Middle Eastern	7 (0.17)	21 (1.68)	28 (0.52)
	Multiracial or multiethnic	102 (2.45)	95 (7.62)	197 (3.64)
**Gender** **(n=5406)**				
	Man	1493 (35.88)	412 (33.09)	1905 (35.24)
	Woman	2589 (62.22)	780 (62.65)	3369 (62.32)
	Nonbinary	17 (0.41)	24 (1.93)	41 (0.76)
	Transgender people	10 (0.24)	8 (0.64)	18 (0.33)
	Other	52 (1.25)	21 (1.69)	73 (1.35)
**Sexual orientation (n=5382)**				
	Straight or heterosexual	3728 (89.96)	1077 (87)	4805 (89.28)
	Lesbian or gay	148 (3.57)	49 (3.96)	197 (3.66)
	Bisexual	226 (5.45)	83 (6.7)	309 (5.74)
	Other	42 (1.01)	29 (2.34)	71 (1.32)
**Age (years; n=5119)**				
	18 to 35	1417 (35.5)	476 (42.24)	1893 (36.98)
	36 to 55	1579 (39.55)	392 (34.78)	1971 (38.5)
	56 to ≥85	996 (24.95)	259 (22.98)	1255 (24.52)
**Household** **i** **ncome (n=5355) **				
	<US $25,000	1029 (24.96)	274 (22.22)	1303 (24.33)
	US $25,000 to $34,999	645 (15.65)	174 (14.11)	819 (15.29)
	US $35,000 to $49,999	638 (15.48)	195 (15.82)	833 (15.56)
	US $50,000 to $74,999	755 (18.32)	240 (19.46)	995 (18.58)
	US ≥$75,000	1055 (25.59)	350 (28.39)	1405 (26.24)
**Educational** **a** **ttainment (n=5404) **				
	Less than high school	221 (5.31)	99 (7.97)	320 (5.92)
	High school or general education diploma	1008 (24.22)	237 (19.08)	1245 (23.04)
	Some college, vocational or technical	1451 (34.86)	295 (23.75)	1746 (32.31)
	Bachelor’s degree	1031 (24.77)	375 (30.19)	1406 (26.02)
	Master’s degree or above	451 (10.84)	236 (19)	687 (12.71)
**Employment (n=5405) **				
	Unemployed or nontraditional work	1819 (43.7)	582 (46.82)	2401 (44.42)
	Nonessential worker	1300 (31.23)	393 (31.62)	1693 (31.32)
	Essential worker	1043 (25.06)	268 (21.56)	1311 (24.26)
**Substance use coping behaviors (n=5404)**				
	Other coping behaviors	3895 (93.63)	1206 (96.94)	5101 (94.39)
	E-cigarette or nicotine vape use	75 (1.8)	8 (0.64)	83 (1.54)
	Increased alcohol use	68 (1.63)	19 (1.53)	87 (1.61)
	Marijuana use	99 (2.38)	10 (0.8)	109 (2.02)
	Illicit substance use	23 (0.55)	1 (0.08)	24 (0.44)

**Table 2:** Substance use before and during the COVID-19 pandemic.

		Before the COVID-19 pandemic (n=5130), n (%)	During the COVID-19 pandemic (n=3802), n (%)
**Tobacco use**			
	Not at all	3418 (67.26)	2612 (69.32)
	Once to several times per month	366 (7.2)	232 (6.16)
	Once to several times per week	358 (7.04)	260 (6.9)
	Every day to several times per day	940 (18.5)	664 (17.62)
**E-cigarette or nicotine vape use**			
	Not at all	4017 (79.31)	3028 (80.7)
	Once to several times per month	363 (7.17)	219 (5.84)
	Once to several times per week	352 (6.95)	269 (7.17)
	Every day to several times per day	333 (6.57)	236 (6.29)
**Alcohol use**			
	Not at all	2140 (42.04)	1625 (43.02)
	Once to several times per month	1563 (30.71)	1051 (27.83)
	Once to several times per week	1083 (21.28)	814 (21.55)
	Every day to several times per day	304 (5.97)	287 (7.6)
**Marijuana use**			
	Not at all	3778 (74.92)	2857 (76.15)
	Once to several times per month	460 (9.12)	313 (8.34)
	Once to several times per week	334 (6.62)	246 (6.56)
	Every day to several times per day	471 (9.34)	336 (8.96)
**Illicit substance use**			
	Not at all	4470 (88.29)	3342 (89.26)
	Once to several times per month	225 (4.44)	136 (3.63)
	Once to several times per week	216 (4.27)	148 (3.95)
	Every day to several times per day	152 (3)	118 (3.15)

**Table 3:** Substance use before and during the COVID-19 pandemic by US-born and foreign-born participants.

		US born (n=5130), n (%)			Foreign born (n=3802), n (%)	
		Before the COVID-19 pandemic	During the COVID-19 pandemic	Before the COVID-19 pandemic		During the COVID-19 pandemic
**Tobacco use**						
	Not at all	2456 (62.93)	1903 (65.35)	962 (81.59)		710 (82.56)
	Once to several times per month	299 (7.66)	191 (6.56)	67 (5.68)		41 (4.77)
	Once to several times per week	302 (7.74)	218 (7.49)	56 (4.75)		42 (4.88)
	Every day to several times per day	846 (21.68)	600 (20.6)	94 (7.97)		67 (7.79)
**E-cigarette or nicotine vape use**						
	Not at all	3017 (77.58)	2277 (78.54)	1000 (85.03)		752 (87.75)
	Once to several times per month	288 (7.41)	183 (6.31)	75 (6.38)		37 (4.32)
	Once to several times per week	298 (7.66)	231 (7.97)	54 (4.59)		39 (4.55)
	Every day to several times per day	286 (7.35)	208 (7.17)	47 (4)		29 (3.38)
**Alcohol use**						
	Not at all	1554 (39.82)	1190 (40.74)	586 (49.37)		436 (50.7)
	Once to several times per month	1207 (30.92)	812 (27.8)	356 (29.99)		242 (28.14)
	Once to several times per week	894 (22.91)	680 (23.28)	189 (15.92)		134 (15.58)
	Every day to several times per day	248 (6.35)	239 (8.18)	56 (4.72)		48 (5.58)
**Marijuana use**						
	Not at all	2761 (71.29)	2107 (72.63)	1017 (86.92)		752 (87.95)
	Once to several times per month	398 (10.28)	273 (9.41)	62 (5.30)		41 (4.8)
	Once to several times per week	294 (7.59)	221 (7.62)	40 (3.42)		26 (3.04)
	Every day to several times per day	420 (10.84)	300 (10.34)	51 (4.36)		36 (4.21)
**Illicit substance use**						
	Not at all	3380 (86.96)	2551 (88.09)	1094 (92.71)		793 (93.07)
	Once to several times per month	181 (4.66)	112 (3.87)	44 (3.72)		24 (2.82)
	Once to several times per week	196 (5.04)	131 (4.52)	20 (1.69)		19 (2.23)
	Every day to several times per day	130 (3.34)	102 (3.52)	22 (1.86)		16 (1.88)

### Differences in Substance Use Before and During the COVID-19 Pandemic

Using the Stuart-Maxwell test of asymptotic symmetry and marginal homogeneity, we found significant differences in alcohol use in the overall sample (Table 4). In Table 5, we see that the largest contribution to *χ^2^* symmetry (*χ^2^*_3_=20.2) was owing to differences in no alcohol use before the COVID-19 pandemic to once to several times per month during the COVID-19 pandemic (ie, a 1.71% change) and once to several times per month before the COVID-19 pandemic to not at all during the COVID-19 pandemic (ie, a 3.37% change). The second largest contribution (*χ^2^*_3_=15.6) was observed with alcohol use once to several times per week before the COVID-19 pandemic to every day to several times per day during the COVID-19 pandemic (ie, a 2.25% change) and every day to several times per day before the COVID-19 pandemic to once to several times per week during to the COVID-19 pandemic (ie, a 1.07% change). All other substance use changes reported before and during the pandemic were not significant but can be found in Multimedia Appendix 1.

Then, we assessed differences in substance use before and during the pandemic by nativity and found them to be significantly different between US-born and foreign-born individuals (Table 5). Among the US-born participants, the largest contribution to the symmetry *χ^2^* (*χ^2^*_3_=13.3) was owing to differences in no alcohol use before the COVID-19 pandemic to once to several times per month during the COVID-19 pandemic (ie, a 1.77% change) and once to several times per month before the COVID-19 pandemic to not at all during the COVID-19 pandemic (ie, a 3.28% change). The second largest contribution among US-born participants (*χ^2^*_3_=13.2) was in alcohol use once to several times per week before the COVID-19 pandemic to every day to several times per day during the COVID-19 pandemic (ie, a 2.22% change) and every day to several times per day before the COVID-19 pandemic to once to several times per week during to the COVID-19 pandemic (ie, a 1.00% change). See Table 6 for more detail.

As seen in Table 7 among foreign-born participants, the largest contribution to the symmetry *χ^2^* (*χ^2^*_3_=7.4) was owing to differences in no prior alcohol use before the COVID-19 pandemic to once to several times per month during the COVID-19 pandemic (ie, a 1.5% change) and once to several times per month before the COVID-19 pandemic to not at all during the COVID-19 pandemic (ie, a 3.7% change). The second largest *χ^2^* contribution was alcohol use once to several times per month to every day to several times a day (*χ^2^*_3_=3.8). Of the 843 respondents, 23 (2.7%) shifted from alcohol use once to several times per month before the pandemic to every day to several times per day during the pandemic, compared with 18 (2.1%) who shifted from alcohol use every day to several times per day before the COVID-19 pandemic to once to several times per month during the COVID-19 pandemic. The third largest contribution (*χ^2^*_3_=2.6) was in alcohol use once to several times per week before the COVID-19 pandemic to every day to several times per day during the COVID-19 pandemic (ie, a 2.4% change) and every day to several times per day before the COVID-19 pandemic to once to several times per week during the COVID-19 pandemic (ie, a 1.3% change). Refer to Table 5 for all *the χ^2^* contributions to symmetry.

**Table 4:** Alcohol use before the COVID-19 pandemic compared with during the pandemic^a^.

Before the COVID-19 pandemic	During the COVID-19 pandemic				
	Not at all, n (∆%)	Once to several times per month, n (∆%)	Once to several times per week, n (∆%)	Every day to several times per day, n (Δ%)	Total
Not at all	1432 (N/A^b^)	64 (1.71)	22 (0.59)	4 (0.11)	1522
Once to several times per month	126 (3.37)	826 (N/A)	136 (3.64)	15 (0.40)	1103
Once to several times per week	29 (0.77)	127 (3.40)	611 (N/A)	84 (2.25)	851
Every day to several times per day	12 (0.32)	21 (0.56)	40 (1.07)	182 (N/A)	255
Total	1599 (N/A)	1038 (N/A)	809 (N/A)	285 (N/A)	3731

^a^Symmetry (asymptotic) was based on *χ^2^*_6_=42.1 and *P*≤.001; marginal homogeneity was based on the Stuart-Maxwell test (*χ^2^*_3_=30.0; *P*≤.001).

^b^N/A: not applicable (as these are the references to compare and contrast contributions to symmetry).

**Table 5:** Contribution to symmetry χ^2^ from alcohol use before and during the COVID-19 pandemic.

Change	Before or during COVID-19	Overall *χ*^2^ (*df*)	US born *χ*^2^ (*df*)	Foreign born *χ*^2^ (*df*)
Not at all	Once to several times per month	20.2 (3)	13.3 (3)	7.4 (3)
Not at all	Once to several times per week	1.0 (3)	0.1 (3)	1.7 (3)
Not at all	Every day to several times per day	4.0 (3)	2.6 (3)	2.0 (3)
Once to several times per month	Once to several times per week	0.3 (3)	0.1 (3)	0.6 (3)
Once to several times per month	Every day to several times per day	1.0 (3)	0.0 (3)	3.8 (3)
Once to several times per week	Every day to several times per day	15.6 (3)	13.2 (3)	2.6 (3)

**Table 6:** Alcohol use before the COVID-19 pandemic compared with during the pandemic among US-born participants^a^.

Before the COVID-19 pandemic	During the COVID-19 pandemic				
	Not at all, n (Δ%)	Once to several times per month, n (Δ%)	Once to several times per week, n (Δ%)	Every day to several times per day, n (Δ%)	Total
Not at all	1049 (N/A^b^)	51 (1.77)	17 (0.59)	4 (0.14)	1121
Once to several times per month	95 (3.28)	630 (N/A)	113 (3.91)	12 (0.42)	850
Once to several times per week	19 (0.66)	109 (3.77)	518 (N/A)	64 (2.22)	710
Every day to several times per day	10 (0.34)	11 (0.38)	29 (1.00)	157 (N/A)	207
Total	1173 (N/A)	801 (N/A)	677 (N/A)	237 (N/A)	2888

^a^Marginal homogeneity based on Stuart-Maxwell *χ^2^*_3_ of 22.6 (*P*>.001).

^b^N/A: not applicable (as these are the references to compare and contrast contributions to symmetry).

**Table 7:** Alcohol use before the COVID-19 pandemic compared with during the pandemic among foreign-born participants^a^.

Before the COVID-19 pandemic	During the COVID-19 pandemic				
	Not at all, n (Δ%)	Once to several times per month, n (Δ%)	Once to several times per week, n (Δ%)	Every day to several times per day, n (Δ%)	Total
Not at all	383 (N/A^b^)	13 (1.5)	5 (0.6)	0 (0)	401
Once to several times per month	31 (3.7)	196 (N/A)	23 (2.7)	3 (0.4)	253
Once to several times per week	10 (1.2)	18 (2.1)	93 (N/A)	20 (2.4)	141
Every day to several times per day	2 (0.2)	10 (1.2)	11 (1.3)	25 (N/A)	48
Total	426 (N/A)	237 (N/A)	132 (N/A)	48 (N/A)	843

^a^Marginal homogeneity based on Stuart-Maxwell test *χ^2^*_3_ of 210.4 (*P*=.02).

^b^N/A: not applicable (as these are the references to compare and contrast contributions to symmetry).

## Discussion

### Principal Findings

We assessed changes in the use of combustible tobacco, e-cigarette and nicotine vape, alcohol, marijuana, and other illicit substances. Although use of alcohol was found to have significant changes before and during the pandemic in our overall sample, we did not observe significant changes in the use of tobacco, e-cigarette and nicotine vape, marijuana, or other illicit substances (Multimedia Appendix 1). Then, we examined alcohol use changes by comparing US-born and foreign-born participants and found significant changes in each group. The *χ^2^* contributions to symmetry indicated that the largest contributors to significant changes in substance use before and during the COVID-19 pandemic were similar for the overall sample in the United States and the US-born sample. Similarities were observed across US-born and foreign-born samples; however, changes in increased alcohol use among these groups were observed on (1) no alcohol use before the pandemic to using alcohol once to several times a month and (2) once to several times per week to every day to several times per day. Increases in alcohol use may indicate maladaptive coping with the effects of social distancing and isolation [8,9]. The increase from weekly alcohol use to daily use indicates increased physiological and psychological risk as well as a risk of developing possible alcohol use disorders [28].

Although we found that significant changes were observed from no prior alcohol use to some level of increased use, we also observed the opposite in both the US-born and foreign-born groups. The decrease in alcohol use was slightly more pronounced among foreign-born participants. That is, there was a 5.1% overall change in some level of alcohol use before the pandemic to no alcohol use during the pandemic among foreign-born participants, compared with a 4.3% change among US-born participants. In our findings, the largest shift was not associated with increased alcohol use but with decreased alcohol use. This decrease in substance use may also be indicative of isolation [26].

### Comparison With Prior Work

The use of both licit and illicit substances can have deleterious effects not only on mental health but also on physiological health and physical functioning, as well as damage organ systems that can increase morbidity and mortality from COVID-19 [29]. First, given our findings, the synergistic effects of isolation and alcohol use must be considered. Although participants may use alcohol to cope with the deleterious effects of isolation and psychological distress caused by the COVID-19 pandemic, the use of substances in themselves has been reported to be maladaptive [9,26]. One could argue that the use of alcohol has social connotations, whereas others would argue that substance use creates isolation owing to its taboo in the social context [26]. In the context of our findings, the increase from weekly alcohol use to daily use may be interpreted as increased personal use, not during social gatherings, given the context of social distancing practices. Conversely, decreases in alcohol use may also be tracked to the social nature of general alcohol consumption that the pandemic disrupted [30,31].

When specifically seeking to understand the differences in alcohol use between US-born and foreign-born participants during the COVID-19 pandemic, the available studies were limited. Overall, when foreign-born immigrants were assessed for substance use during the pandemic, they reported less substance use when compared with their US-born counterparts [24,26,27]. Our findings parallel what the limited studies have found, yet we did find that changes in decreased alcohol use were higher in foreign-born individuals compared with US-born individuals, that is, from once to several times per month to not at all during the pandemic.

Nevertheless, decreased alcohol use may be observed in our sample owing to a wide range of socioeconomic status factors such as low income and unemployment and environmental factors such as access and scarcity [32]. This may also be an artifact of our oversampling of lower socioeconomic status groups. Regardless, more information is needed to assess the deleterious effects on physical and mental health based on the frequency and number of drinks consumed as well as the level of risk of developing possible alcohol use disorders.

Our findings at this stage may indicate small percentage changes but may reveal the ideal point of intervention to mitigate the effect of use disorders. Moreover, we must acknowledge the synergetic effects of mental health and physiological health, especially in the context of COVID-19. As such, the extremes of general alcohol consumption and use disorders must continue to be monitored not only for concomitant health effects, such as alcohol-associated liver disease and mortality [28,29], but also to better understand the social and environmental effects of the COVID-19 pandemic and the policies that affect changes in alcohol use [33]. Future research should focus on polysubstance, mental health, coping behaviors, and prior substance use to identify the groups most at risk and design the most appropriate intervention strategies.

### Limitations

Our study had some limitations. First, although the survey was anonymous, the possibility of bias in recall and responses must be considered. There is a possible recall bias from self-reports, as we asked about behaviors before COVID-19 owing to the data collection starting in May 2021 and ending in January 2022. This may be magnified by asking sensitive questions concerning substance use behaviors before and during the pandemic, increasing the response bias. Second, the data were obtained from a cross-sectional survey that provided a descriptive analysis of substance use change in a large US sample. However, this sample is not representative of the United States. Moreover, because this was a cross-sectional sample, we could not discern causality or temporal directionality. The data and descriptive analysis provided a solid foundation for further examination and identification of substance use patterns across a diverse US sample.

### Conclusions

Pandemics are predicted to increase in frequency in the near future. To better prepare for the indirect effects of public health policies meant to protect the health of individuals, we must also prepare for their indirect effects on mental health and related coping mechanisms. Substance use affects both physical and mental health and will therefore require a multimodal approach to efficiently and effectively address and intervene on the deleterious effects, especially for underserved and underrepresented communities.

## Appendix

Multimedia Appendix 1 Full study comparisons of substance use prior to and during the pandemic.

## Abbreviations

NIH: National Institutes of Health
