# Supplementary material for: Substance Use From Social Distancing and Isolation by US Nativity During the Time of COVID-19: Cross-sectional Study
Source: JMIR Public Health Surveill. 2023 Feb 17;9:e38163. doi: 10.2196/38163 (PMC10473437; doi:10.2196/38163)
Supplement: Multimedia Appendix 1 [file publichealth_v9i1e38163_app1.docx]

Appendix

| Table X. Tobacco use prior to Covid-19 pandemic compared to during pandemic | | | | | | | | | | |
| --- | --- | --- | --- | --- | --- | --- | --- | --- | --- | --- |
|  | During Covid-19 Pandemic | | | | | | | | |  |
|  | Not at all | | Once to several times per month | | Once to several times per week | | Every day to several times per day | |  |  |
| Prior to Covid-19 Pandemic | n | (∆%) | n | (∆%) | n | (∆%) | n | (∆%) | Total |  |
| Not at all | 2,519 | -- | 31 | 0.8 | 9 | 0.2 | 11 | 0.3 | 2,570 |  |
| Once to several times per month | 30 | 0.8 | 139 | -- | 54 | 1.4 | 22 | 0.6 | 245 |  |
| Once to several times per week | 9 | 0.2 | 45 | 1.2 | 152 | -- | 46 | 1.2 | 252 |  |
| Every day to several times per day | 35 | 0.9 | 18 | 0.5 | 43 | 1.1 | 590 | -- | 686 |  |
| Total | 2,593 | | 233 | | 258 | | 669 | | 3,753 |  |
| Notes. Marginal homogeneity based on Stuart-Maxwell test *x*^2^(*df*=3)=4.81, *p*=.18 | | | | | | | | | | |

| Table X. E-cigarette and/or vaporizer use prior to Covid-19 pandemic compared to during pandemic | | | | | | | | | |
| --- | --- | --- | --- | --- | --- | --- | --- | --- | --- |
|  | During Covid-19 Pandemic | | | | | | | | |
|  | Not at all | | Once to several times per month | | Once to several times per week | | Every day to several times per day | |  |
| Prior to Covid-19 Pandemic | n | (∆%) | n | (∆%) | n | (∆%) | n | (∆%) | Total |
| Not at all | 2,947 | -- | 31 | 0.8 | 19 | 0.5 | 12 | 0.3 | 3,009 |
| Once to several times per month | 40 | 1.1 | 139 | -- | 46 | 1.2 | 12 | 0.3 | 237 |
| Once to several times per week | 8 | 0.2 | 37 | 1.0 | 172 | -- | 37 | 1.0 | 254 |
| Every day to several times per day | 14 | 0.4 | 11 | 0.3 | 32 | 0.9 | 178 | -- | 235 |
| Total | 3,009 | | 218 | | 269 | | 239 | | 3,735 |
| Notes. Marginal homogeneity based on Stuart-Maxwell test *x*^2^(*df*=3)=2.66, *p*=.45 | | | | | | | | | |

| Table X. Marijuana use prior to Covid-19 pandemic compared to during pandemic | | | | | | | | | |
| --- | --- | --- | --- | --- | --- | --- | --- | --- | --- |
|  | During Covid-19 Pandemic | | | | | | | | |
|  | Not at all | | Once to several times per month | | Once to several times per week | | Every day to several times per day | |  |
| Prior to Covid-19 Pandemic | n | (∆%) | n | (∆%) | n | (∆%) | n | (∆%) | Total |
| Not at all | 2,784 | -- | 44 | 1.2 | 12 | 0.3 | 10 | 0.3 | 2,850 |
| Once to several times per month | 31 | 0.8 | 220 | -- | 62 | 1.7 | 18 | 0.5 | 331 |
| Once to several times per week | 9 | 0.2 | 29 | 0.8 | 137 | -- | 57 | 1.5 | 232 |
| Every day to several times per day | 11 | 0.3 | 21 | 0.6 | 32 | 0.9 | 252 | -- | 316 |
| Total | 2,835 | | 314 | | 243 | | 337 | | 3,729 |
| Notes. Marginal homogeneity based on Stuart-Maxwell test *x*^2^(*df*=3)=7.13, *p*=.07 | | | | | | | | | |

| Table X. Illicit substance use prior to Covid-19 pandemic compared to during pandemic | | | | | | | | | |
| --- | --- | --- | --- | --- | --- | --- | --- | --- | --- |
|  | During Covid-19 Pandemic | | | | | | | | |
|  | Not at all | | Once to several times per month | | Once to several times per week | | Every day  to several times per day | |  |
| Prior to Covid-19 Pandemic | n | (∆%) | n | (∆%) | n | (∆%) | n | (∆%) | Total |
| Not at all | 3,286 | -- | 20 | 0.5 | 9 | 0.2 | 5 | 0.1 | 3,320 |
| Once to several times per month | 19 | 0.5 | 83 | -- | 24 | 0.6 | 21 | 0.6 | 147 |
| Once to several times per week | 8 | 0.2 | 22 | 0.6 | 93 | -- | 29 | 0.8 | 152 |
| Every day to several times per day | 7 | 0.2 | 10 | 0.3 | 26 | 0.7 | 63 | -- | 106 |
| Total | 3,320 | | 135 | | 152 | | 118 | | 3,725 |
| Notes. Marginal homogeneity based on Stuart-Maxwell test *x*^2^(*df*=3)=, *p*=.54 | | | | | | | | | |
